# Comparative Effectiveness of Hyaluronidase Injections in Carpal Tunnel Syndrome: A Systematic Review and Meta-Analysis

**DOI:** 10.7759/cureus.111912

**Published:** 2026-07-01

**Authors:** Satyasheel S Asthana, Arvind K Sharma, Mohit K Srivastava, Ravi Gaur

**Affiliations:** 1 Physical Medicine and Rehabilitation, All India Institute of Medical Sciences, Raebareli, IND; 2 Physical Medicine and Rehabilitation, Vardhman Mahavir Medical College and Safdarjung Hospital, New Delhi, IND; 3 Physical Medicine and Rehabilitation, All India Institute of Medical Sciences, Jodhpur, IND

**Keywords:** boston carpal tunnel syndrome questionnaire, carpal tunnel syndome, hyaluronidase, nerve conduction studies (ncs), systematic review and meta-analysis

## Abstract

Carpal tunnel syndrome (CTS) is a common entrapment neuropathy associated with pain, sensory disturbances, functional impairment, and reduced quality of life. Hyaluronidase, an enzyme capable of degrading hyaluronic acid and facilitating perineural hydrodissection, has emerged as a potential minimally invasive treatment option for CTS.

This systematic review and meta-analysis evaluated the effectiveness of hyaluronidase injections in CTS compared with other injection therapies. This Preferred Reporting Items for Systematic Reviews and Meta-Analyses (PRISMA)-compliant systematic review and meta-analysis was prospectively registered in the International Prospective Register of Systematic Reviews (PROSPERO) (CRD420251046156). PubMed, Scopus, and the Cochrane Central Register of Controlled Trials (CENTRAL) were searched, supplemented by Google Scholar, Ovid Discovery, and ClinicalKey. Randomized controlled trials comparing hyaluronidase injections with control interventions in adults with CTS were included. Primary outcomes included symptom severity scale (SSS), functional status scale (FSS), sensory nerve conduction velocity (SNCV), and distal motor latency (DML). Secondary outcomes included visual analogue scale (VAS) and cross-sectional area (CSA) of the median nerve. Random-effects meta-analysis was performed using IBM SPSS Statistics for Windows, Version 31.0 (IBM Corp., Armonk, New York, United States). Risk of bias and certainty of evidence were assessed using the revised Cochrane Risk of Bias tool for randomized trials (RoB 2) and the Grading of Recommendations Assessment, Development and Evaluation (GRADE) approach, respectively.

Four randomized controlled trials involving 230 participants were included. Primary pooled analyses demonstrated directional trends favoring hyaluronidase for SSS at three months (MD=-0.17; 95% CI=-0.65 to 0.31) and six months (MD=-0.42; 95% CI=-1.29 to 0.45), FSS at three months (MD=-0.23; 95% CI=-0.95 to 0.49) and six months (MD=-0.30; 95% CI=-1.13 to 0.54), DML at three months (MD=-0.21 ms; 95% CI=-0.68 to 0.25) and six months (MD=-0.55 ms; 95% CI=-1.14 to 0.03), and SNCV at three months (MD=0.56 m/s; 95% CI=-3.57 to 4.69) and six months (MD=2.23 m/s; 95% CI=-2.88 to 7.34); however, none of these primary outcomes reached statistical significance. Pooled analysis demonstrated a statistically significant reduction in VAS scores at six months favoring hyaluronidase (MD=-1.97; 95% CI=-3.88 to -0.06; p=0.04). Subgroup analyses demonstrated significantly greater improvement with hyaluronidase compared with dexamethasone for SSS, FSS, and DML at the six-month follow-up. Considerable heterogeneity was observed across most pooled analyses, and sensitivity analyses identified one study as the principal contributor to heterogeneity. The certainty of evidence for primary outcomes was low.

Current evidence does not demonstrate a consistent or statistically robust benefit of hyaluronidase injection in improving symptom severity, functional status, DML, and SNCV in CTS, although directional trends and subgroup findings suggest potential benefit in specific comparisons, particularly against dexamethasone. Further high-quality, adequately powered randomized controlled trials with standardized outcome measures are required to establish its effectiveness.

## Introduction and background

Carpal tunnel syndrome (CTS) is a highly prevalent entrapment neuropathy of the upper limb. The global pooled prevalence is about 14%, although estimates vary across different populations [1]. CTS is characterized by compression of the median nerve as it passes beneath the transverse carpal ligament within the rigid, fibro-osseous carpal tunnel [1]. CTS is increasingly recognized as a disorder involving subsynovial connective tissue fibrosis and impaired median nerve mobility rather than solely a compressive neuropathy. Histopathological and biomechanical studies have demonstrated fibrosis, adhesion formation, and restriction of normal median nerve excursion within the carpal tunnel [2].

Its etiopathogenesis is multifactorial and involves a complex interplay of increased intracarpal pressure, perineural fibrosis, synovial hypertrophy of the flexor tendons, impaired microcirculation, and progressive ischemic injury to the median nerve, ultimately resulting in demyelination and axonal dysfunction [3,4]. Several risk factors predispose individuals to CTS, including repetitive hand use, forceful or vibratory occupational activities, female sex, diabetes mellitus, obesity, hypothyroidism, pregnancy, rheumatoid arthritis, and anatomical variations that reduce tunnel volume [5].

CTS causes pain, numbness, and tingling in the median nerve distribution, often progressing to weakness, reduced or altered sensation, and reduced grip strength. It significantly impairs quality of life by disturbing sleep, fine motor activities, and daily productivity. These limitations, ranging from difficulty in household chores to reduced work efficiency, often lead to job loss, anxiety, and depression [6-9]. As a result, CTS imposes both personal and socioeconomic burden, underscoring the importance of early diagnosis and effective, long-lasting interventions [7,8].

The diagnosis of CTS is established through a variety of clinical features. History and physical examination provide diagnostic clues, and electrodiagnostic studies confirm it [10]. Ultrasound assessment of the carpal tunnel has also emerged as a valuable tool in aiding in the diagnosis of CTS. Ultrasound can also assess nerve echogenicity, vascularity, mobility, and cross-sectional area (CSA) of the median nerve [11-13].

CTS management spans a broad therapeutic spectrum, starting with non-pharmacological interventions such as ergonomic modifications, wrist splints, therapeutic exercises, and lifestyle modifications [14,15]. Pharmacological treatments include oral analgesics, non-steroidal anti-inflammatory drugs, neuropathic pain agents, and short-term use of oral corticosteroids for symptomatic relief [16]. Injection therapy plays a central role in modern CTS management and encompasses a variety of perineural or intra-tunnel injectates [16,17]. For patients with severe CTS or those who fail conservative and injection-based therapies, surgical decompression remains the definitive option. Surgical approaches range from ultrasound-guided percutaneous release to open carpal tunnel release [18-20].

Minimally invasive management or injection-based therapies include various injectates in the carpal tunnel. Ultrasound-guided perineural injection techniques have gained popularity in recent years. Several pharmacologic and biologic injectates have been investigated for CTS, including corticosteroids, 5% dextrose, platelet-rich plasma, and hyaluronidase [20-29]. Few studies also investigated botulinum toxin A [30] and midazolam [27], although both are not commonly used in CTS. Another approach is median nerve hydrodissection using injectable solutions (like corticosteroids, platelet-rich plasma, prolotherapy, and hyaluronidase) to mechanically separate nerves from surrounding tissue reducing compressive forces [31].

Hyaluronidase, an enzyme that degrades hyaluronic acid in the extracellular matrix, has been explored as an adjuvant to perineural injections to enhance tissue permeability and facilitate hydrodissection. Several studies have investigated the use of hyaluronidase alone or in combination with local anesthetics, corticosteroids, or other agents in the management of mild to moderate CTS [25-29]. Although corticosteroids are widely used in CTS management, their benefits are short-lived and may be associated with systemic risk [16].

Despite increasing interest in hyaluronidase injections for CTS, the available evidence remains fragmented, with variability in injectates, comparators, and reported outcomes. This systematic review and meta-analysis evaluated the effect of hyaluronidase injections on functional outcomes and nerve conduction velocity (NCV) parameters in patients with CTS compared to other comparators. Furthermore, the review also reported the adverse events associated with the use of hyaluronidase injections in the treatment of CTS.

## Review

Methods

This systematic review and meta-analysis was conducted following the Preferred Reporting Items for Systematic Reviews and Meta-Analyses (PRISMA) 2020 guidelines [32]. The study was registered prospectively in the International Prospective Register of Systematic Reviews (PROSPERO) (CRD420251046156) on May 15, 2025 [33].

Inclusion and Exclusion Criteria

The inclusion criteria were as follows: adults (≥18 years) diagnosed with CTS based on electrodiagnostic criteria (population); injection of hyaluronidase, with or without adjunctive agents (local anesthetics, corticosteroids, etc.) (intervention); corticosteroid injection, normal saline (placebo), or any other established injection therapy in CTS (comparator); and randomized controlled trials (RCTs) must have used NCV parameters for comparisons or symptom severity scale (SSS) and functional status scale (FSS) of the Boston Carpal Tunnel Questionnaire (BCTQ) or both (outcomes). In addition, RCTs must report outcomes of at least three months of follow-up. The study design was restricted to published RCTs, and the language was limited to articles available in English.

Studies on animal models or in vitro experiments were excluded. Additionally, abstracts, posters, dissertations, unpublished data, and other non-randomized studies were not considered. Observational studies, case reports, and review articles were also excluded. Studies that did not report specific outcomes related to CTS or had incomplete outcome data were omitted. Furthermore, studies involving pediatric patients, post-surgical CTS cases, or CTS secondary to systemic diseases such as rheumatoid arthritis or hypothyroidism, as well as CTS resulting from trauma, were excluded unless they reported separate subgroup data specifically for idiopathic CTS.

Primary outcomes were electrophysiological and questionnaires. Electrophysiological outcomes included sensory nerve conduction velocity (SNCV) and distal motor latency (DML) reflecting median nerve function. Questionnaires included SSS and FSS of BCTQ. Visual analogue scale (VAS) and CSA of the median nerve were secondary outcome measures.

Database and Search

A comprehensive search strategy was designed to ensure the identification of all eligible RCTs on hyaluronidase injections in CTS. Only published studies were sought, with no restrictions on date of publication, although the review was limited to articles in English. The main bibliographic databases searched were PubMed, Scopus, and the Cochrane Central Register of Controlled Trials (CENTRAL). Supplementary searches were also performed in Google Scholar, Ovid Discovery, and ClinicalKey to identify additional potentially relevant studies not indexed in the primary databases. The last date of search was July 31, 2025.

An advanced search strategy was used using Medical Subject Headings (MeSH) terms or keywords based on the database searched (“Carpal Tunnel Syndrome” OR “CTS” OR “Median Nerve Entrapment”) AND (“Hyaluronidase” OR “Hyalase”). Supplementary methods such as citation tracking (snowballing) and checking the reference lists of the included studies were used to identify additional trials. The detailed search syntax for all databases is provided in the Appendices.

Study Selection

Screening of studies by titles and abstracts was carried out independently by two authors. Full texts of potentially eligible studies were retrieved and assessed against the inclusion and exclusion criteria. Any discrepancies were resolved through discussion with the third and fourth authors to reach a consensus.

Data Extraction

Data extraction was done independently by two authors using a standardized Microsoft Excel-based data extraction sheet (Microsoft Corporation, Redmond, Washington, United States). Extracted information included study characteristics, participant demographics, diagnostic criteria, interventions, outcome measures, information required in risk of bias and GRADEpro Guideline Development Tool (GDT) assessment, and reported adverse events or side effects. When a study included multiple eligible intervention arms, these arms were mathematically combined into a single group in accordance with Cochrane Handbook guidance for continuous outcomes [34]. Information on funding sources and reported conflicts of interest of the included studies were also examined. When outcome data were unclear or incompletely reported, corresponding authors were contacted for clarification. Data were extracted directly from published tables and text. No outcome data were estimated from graphical representations. Studies were excluded only when outcome data necessary for quantitative synthesis could not be obtained from the publication or from author correspondence.

Risk of Bias Assessment

The methodological quality of the included studies was independently appraised using the revised Cochrane Risk of Bias tool for randomized trials (RoB 2). Each study was evaluated through five domains (randomization process, deviation from intended intervention, missing outcome data, measurement of outcomes, and selection of reported results). Each domain was graded as low risk of bias, some concerns, and high risk of bias [35-37]. Discrepancies in RoB 2 evaluation were addressed by the authors through a mutual agreement.

Certainty of Evidence

The overall certainty of evidence was assessed for primary outcomes using the Grading of Recommendations Assessment, Development and Evaluation (GRADE) approach implemented through the GRADEpro GDT software. The GRADE framework provides a structured process for rating the certainty of evidence and strength of recommendations. A summary of findings table was generated using the GRADEpro GDT software [38,39].

Statistical Analysis

Data synthesis was done using a random-effects model. Effect sizes of outcomes (all continuous variables) were calculated as mean difference (MD) with corresponding 95% confidence interval (CI). The individual participant will be considered the unit of analysis. For studies including patients with bilateral CTS, both hands will be analyzed as independent observations. Analyses were stratified by follow-up timepoints (three and six months). Random-effects model was used for all analyses as heterogeneity was expected across the included trials (variation in comparators, injection doses, and volumes). Heterogeneity was assessed using the I² statistic and interpreted in accordance with Cochrane Handbook guidance, considering values around 0-40% as might not be important, 30-60% as moderate, 50-90% as substantial, and >75% as considerable, with interpretation informed by clinical and methodological context [34]. Subgroup and sensitivity analyses were also done due to considerable heterogeneity. All statistical analyses were conducted using IBM SPSS Statistics for Windows, Version 31.0 (IBM Corp., Armonk, New York, United States). A p-value of less than 0.05 was considered statistically significant. Forest plots and heterogeneity statistics were generated using custom SPSS syntax commands (random-effects meta-analysis along with the restricted maximum likelihood estimator for between-study variance (τ²); MDs with 95% CI were calculated for continuous outcomes). Egger's regression analysis for assessing publication bias was planned but not done due to a smaller number of studies. Studies with insufficient or non-extractable summary data were excluded from quantitative synthesis for that outcome.

Results

**Figure 1:**
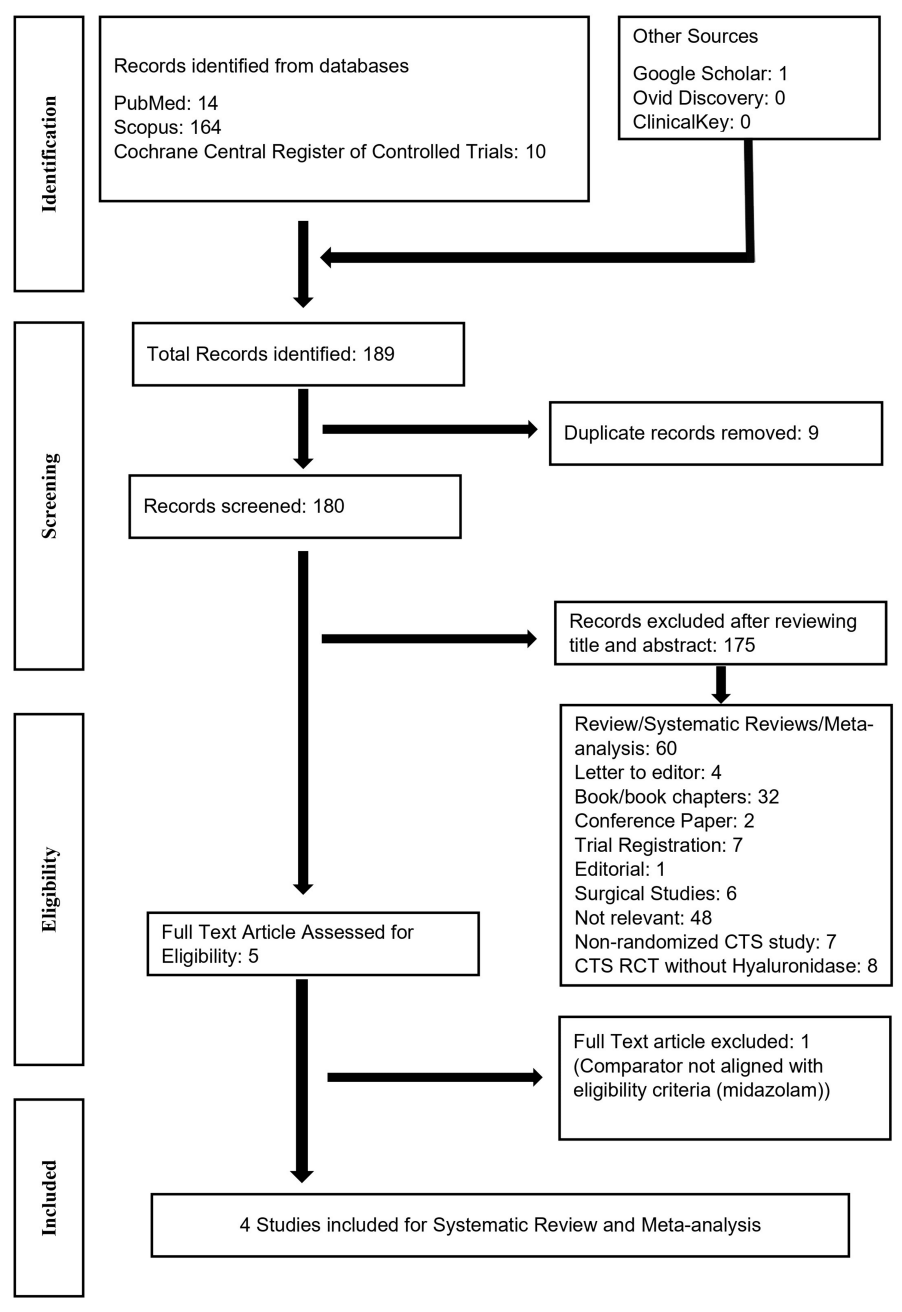
PRISMA flow diagram depicting the study selection process for the systematic review and meta-analysis The PRISMA flow diagram summarizes the process of study identification, screening, eligibility assessment, and inclusion for this systematic review and meta-analysis. After duplicate removal, records were screened based on titles and abstracts, followed by full-text assessment of potentially eligible studies. Studies not meeting the predefined inclusion criteria were excluded with reasons documented. PRISMA: Preferred Reporting Items for Systematic Reviews and Meta-Analyses; CTS: carpal tunnel syndrome; RCT: randomized controlled trial

After a comprehensive search, 189 records were identified. After removing nine duplicates using Zotero (Corporation for Digital Scholarship, Vienna, Virginia, United States) followed by manual verification by two authors, 180 records were screened based on title and abstract. Five reports were identified for full-text assessment [25-29]. One study was excluded, which compared midazolam with hyalase [27]. Midazolam is not a recognized or commonly used agent in CTS and did not align with the predefined eligibility criteria of this review. Finally, four studies were included in the systematic review and meta-analysis [25,26,28,29]. Details of the study selection process are shown in the flow diagram in Figure 1.

Characteristics of the Included Studies

Four RCTs encompassing a total of 230 participants with mild to moderate CTS were included in the systematic review and meta-analysis [25,26,28,29]. The studies were prospective RCTs published between 2019 and 2025. All studies reported randomized allocation methods. Across all studies, CTS diagnosis was established using a combination of clinical assessment and electrodiagnostic criteria [25,26,28,29]. Characteristics of the included studies are shown in Table 1.

**Table 1:** Characteristics of the included RCTs evaluating hyaluronidase injections in CTS This table summarizes the methodological characteristics, sample size, diagnostic criteria, outcome measures, and follow-up duration of the RCTs included in the systematic review and meta-analysis. CTS: carpal tunnel syndrome; RCT: randomized controlled trial; BCTQ: Boston Carpal Tunnel Questionnaire; SSS: symptom severity scale; FSS: functional status scale; SNCV: sensory nerve conduction velocity; DML: distal motor latency; MN CSA: median nerve cross-sectional area; VAS: visual analogue scale; PSL: peak sensory latency; SL: sensory latency; NCV: nerve conduction velocity

Study (year)	Design	Randomization method	Blinding	Sample size	Outcomes assessed	Diagnostic criteria	Follow-up
Alsaeid (2019) [25]	RCT	Computer-generated randomization with allocation concealment (sealed opaque envelopes)	Double-blinded	40	BCTQ (SSS, FSS), SNCV, DML, MN CSA	Clinical + electrodiagnostic, ultrasound	Baseline, 1 week, and 1, 3, and 6 months
Elawamy et al. (2020) [26]	RCT	Computer-generated (web-based) randomization	Double-blinded	60	VAS, Modified BCTQ (Arabic), DML, SNCV, PSL, MN CSA, positive power Doppler	Clinical + electrodiagnostic	Baseline, 1 week, and 1, 3, and 6 months
Elbayomy et al. (2025) [29]	RCT	Computer-generated randomization with allocation concealment (sealed envelopes)	Double-blinded	40	BCTQ (SSS, FSS), SNCV, DML, MN CSA	Clinical + electrophysiology + ultrasound	Baseline, 1 week, and 1, 3, and 6 months
Hussain et al. (2025) [28]	RCT	Computer-generated randomization with allocation concealment (sealed envelopes)	Single-blinded	90	VAS, BCTQ (SSS, FSS), NCV, DML, SL, MN CSA	Clinical + electrodiagnostic	Baseline, 1 week, and 1, 3, and 6 months

Participants were predominantly female. Interventions were delivered under ultrasound guidance in all studies, most commonly using an in-plane ulnar approach, with injectate volumes ranging from 5 mL to 10 mL. The hyaluronidase dose used in Elawamy et al.'s study [26] was 150 IU/mL, while Alsaeid's study [25], Hussain et al.'s study [28], and Elbayomy et al.'s study [29] used 300 IU/mL. It was either injected alone in saline or combined with local anesthetic, while comparator groups received dexamethasone, 5% dextrose, and normal saline [25,26,28,29]. Mild to moderate CTS was defined according to the severity criteria used in the original studies, including established electrodiagnostic grading systems (e.g., Bland or Padua classifications) or equivalent nerve conduction abnormalities characterized by impaired median sensory conduction with or without the prolongation of DML [25,26,28,29].

Laterality of CTS was inconsistently reported across the included studies. Hussain et al. [28] reported 55 right- and 35 left-sided CTS cases among 90 participants, while Elbayomy et al. [29] reported 29 right- and 11 left-sided CTS cases among 40 participants. Alsaeid [25] and Elawamy et al. [26] did not report laterality; however, Elawamy et al. [26] stated that only the more severely affected wrist was injected in patients with bilateral CTS. Details of the intervention and population characteristics are shown in Table 2.

**Table 2:** Baseline demographic characteristics, injection techniques, and adverse events of the included studies This table summarizes participant demographics, CTS severity, intervention protocols, injection techniques, ultrasound-guided approaches, and reported adverse events in the randomized controlled trials included in the systematic review and meta-analysis. CTS: carpal tunnel syndrome; SD: standard deviation; DX: dexamethasone; HD: hyaluronidase; D5%: 5% dextrose; NS: normal saline; US: ultrasound; IU: international units; G: gauge

Study (year)	Groups	CTS grade		Age (mean±SD) (years)	Duration of disease (months)	Gender		Intervention	Total volume (mL)	Needle size	US guidance	Approach	Side effects/adverse events
		Mild	Moderate			Male	Female						
Alsaeid (2019) [25]	DX	10	10	40.18±10.5	Not reported	9	11	3 mL 0.5% bupivacaine + dexamethasone 8 mg (2 mL)	5	23G	Yes	In-plane ulnar approach	Observed patient for 30 minutes post-injection for possible side effects; no side effects
	HD	11	9	42.76±8.3		10	10	3 mL 0.5% bupivacaine + 2 mL saline containing hyaluronidase 300 IU					
Elawamy et al. (2020) [26]	HD	Included mild to moderate but data not reported		40.7±6.5	8.5±2.6	13	13	Hyalase 1500 IU dissolved in 10 mL normal saline	10	26G	Yes	In-plane ulnar approach	Observed patient for 10 minutes for paresthesia/bleeding; monitored for worsening symptoms; no side effects
	Saline			38.3±5.4	8.5±2.1	13	17	10 mL normal saline					
Elbayomy et al. (2025) [29]	D5%	7	13	37.15±6.10	13.05±3.55	6	14	3 mL 0.5% bupivacaine + 7 mL 5% dextrose	10	26G	Yes	Ulnar approach	Observed patient for 30 minutes after injection for any adverse reaction; absence or presence of any side effects not reported during follow-up
	HD	6	14	36.55±7.39	11.65±3.96	5	15	3 mL 0.5% bupivacaine + 7 mL saline containing hyaluronidase 300 IU					
Hussain et al. (2025) [28]	DX	12	18	54.7±10.14	22.5±5.82	Majority females; no data reported		2 mL 0.5% bupivacaine + 1 mL dexamethasone (4 mg/mL) + 2 mL saline	5	22G	Yes	Not reported	Adverse events monitored; absence or presence of any side effects not reported
	D5%	13	17	54.7±12.81	18.2±4.50			5 mL 5% dextrose					
	HD	08	22	55.5±9.23	21.4±6.32			Hyaluronidase 1500 IU in 5 mL saline					

Alsaeid compared hyaluronidase with dexamethasone, and each group contained 20 patients [25]. Elawamy et al. compared hyaluronidase with normal saline, and 60 patients were randomized equally into two groups [26]. Both these studies showed that hyaluronidase significantly improved BCTQ and NCV parameters [25,26]. Elbayomy et al. compared hyaluronidase with 5% dextrose with 20 CTS patients in each group. Although both groups showed significant improvement in BCTQ and NCV parameters, outcomes were more favorable in the 5% dextrose group [29]. Hussain et al. randomized 90 patients equally into three groups (dexamethasone, 5% dextrose, and hyaluronidase), and the hyaluronidase group showed significant improvement in BCTQ and NCV parameters [28].

Risk of Bias Assessment

**Figure 2:**
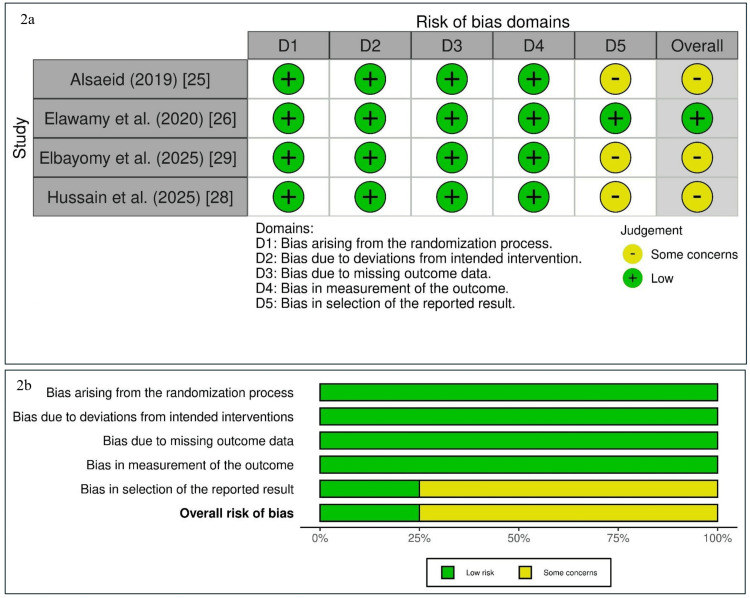
Traffic light plot and summary plot of risk of bias assessment for the included studies (a) Traffic light plot presenting the domain-level and overall risk of bias assessments for the included randomized controlled trials using the revised Cochrane Risk of Bias tool for randomized trials (RoB 2). (b) Corresponding summary plot showing the proportion of studies judged as low risk or having some concerns across each risk of bias domain and overall assessment. D1: bias arising from the randomization process; D2: bias due to deviations from intended interventions; D3: bias due to missing outcome data; D4: bias in measurement of the outcome; D5: bias in selection of the reported result

Risk of bias assessment using RoB 2 indicated that all four included trials were judged as having either low risk of bias or some concerns across the assessed domains. All studies were rated as low risk of bias arising from the randomization process, deviations from intended interventions, and missing outcome data [25,26,28,29]. Additionally, three trials [25,28,29] demonstrated some concerns regarding bias in selection of the reported result, largely attributable to the absence of prospective trial registration. Only one trial [26] was assessed as low risk of bias across all domains. Overall risk of bias assessment indicated some concerns in three studies, with no study classified as high risk [25,28,29]. The RoB 2 traffic light and summary plots are presented in Figure 2.

SSS and FSS

The SSS of BCTQ was reported in three RCTs at three and six months evaluating the effect of hyaluronidase injection compared with control interventions. Considerable heterogeneity was observed among the included studies at three months (I^2^=97%; τ²=0.17) and six months (I^2^=98%; τ²=0.58). The pooled analysis demonstrated that point estimates were in favor of hyaluronidase but no significant improvement in SSS was observed at both three-month (MD=-0.17; 95% CI=-0.65 to 0.31; p=0.49) and six-month (MD=-0.42; 95% CI=-1.29 to 0.45; p=0.35) follow-ups.

The FSS of BCTQ was reported in three RCTs at three and six months evaluating the effect of hyaluronidase injection compared with control interventions. Considerable heterogeneity was observed among the included studies at three months (I^2^=98%; τ²=0.39) and six months (I^2^=99%; τ²=0.53). The pooled analysis demonstrated a non-significant trend favoring hyaluronidase but did not reach statistical significance at both three months (MD=-0.23; 95% CI=-0.95 to 0.49; p=0.53) and six months (MD=-0.3; 95% CI=-1.13 to 0.54; p=0.49).

**Figure 3:**
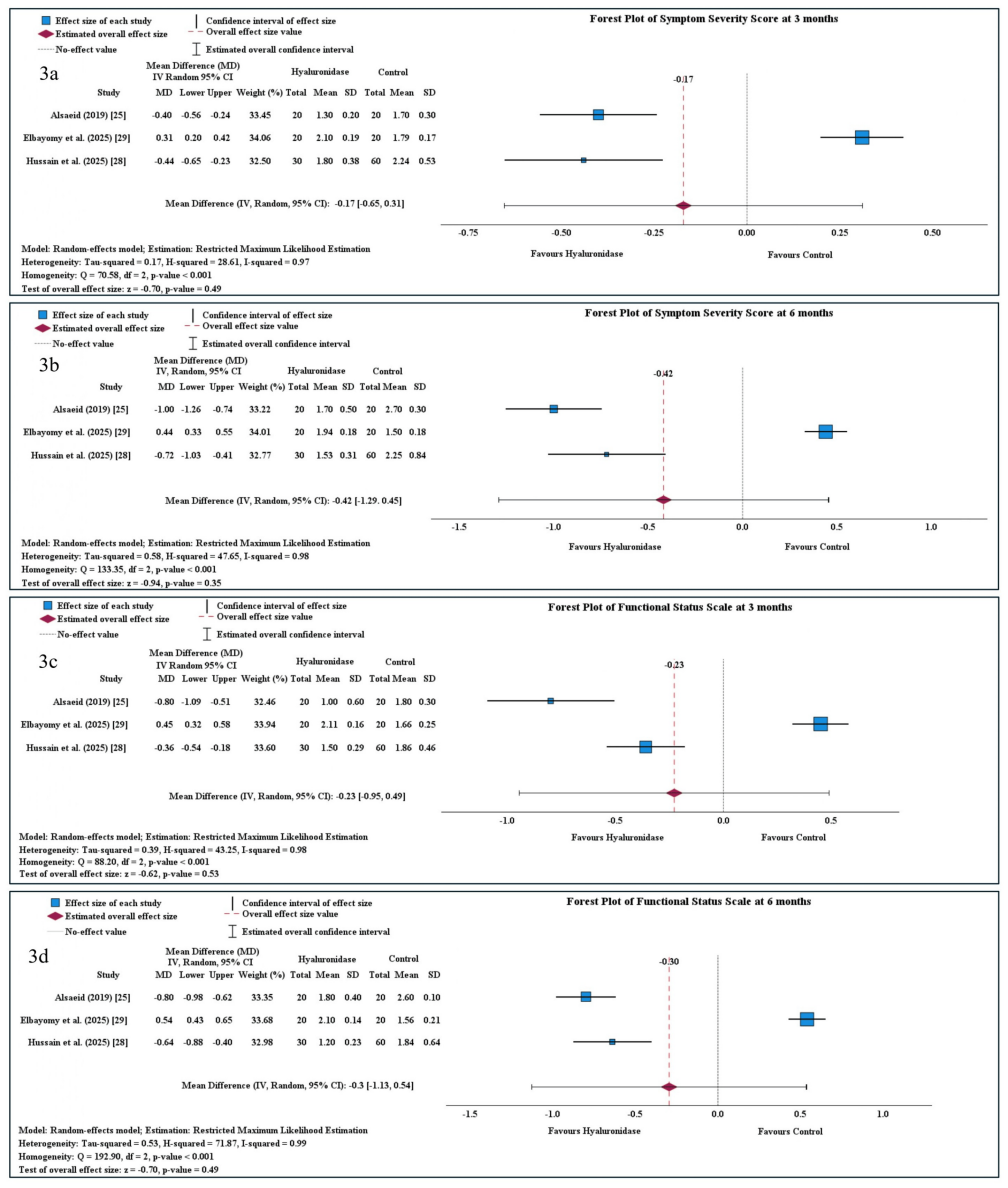
Forest plots comparing hyaluronidase with control interventions for symptom severity score and functional status scale at three and six months Forest plots comparing hyaluronidase injections with control interventions for Boston Carpal Tunnel Questionnaire outcomes using random-effects meta-analysis. (a) Forest plot for symptom severity score at three months. (b) Forest plot for symptom severity score at six months. (c) Forest plot for functional status scale at three months. (d) Forest plot for functional status scale at six months. MD: mean difference; CI: confidence interval; IV: inverse variance; SD: standard deviation

Details of the analysis of SSS and FSS at three and six months are shown in the forest plot in Figure 3.

DML and SNCV

The DML was reported in three RCTs at three months and all four RCTs at six months evaluating the effect of hyaluronidase injection compared with control interventions. Considerable heterogeneity was observed among the included studies at three months (I^2^=94%; τ²=0.15) and six months (I^2^=96%; τ²=0.33). At three and six months, hyaluronidase injection in CTS is associated with a trend towards improvement of DML. However, these effects did not reach statistical significance at three months (MD=-0.21 ms; 95% CI=-0.68 to 0.25; p=0.37) and six months (MD=-0.55 ms; 95% CI=-1.14 to 0.03; p=0.07).

The SNCV was reported in three RCTs at three months and all four RCTs at six months evaluating the effect of hyaluronidase injection compared with control interventions. Considerable heterogeneity was observed among the included studies at three months (I^2^=99%; τ²=12.90) and six months (I^2^=99%; τ²=26.42). At three and six months, hyaluronidase injection in CTS was associated with a directional trend towards improvement of SNCV. However, these effects did not reach a statistically significant improvement at three months (MD=0.56 m/s; 95% CI=-3.57 to 4.69; p=0.79) and six months (MD=2.23 m/s; 95% CI=-2.88 to 7.34; p=0.39).

**Figure 4:**
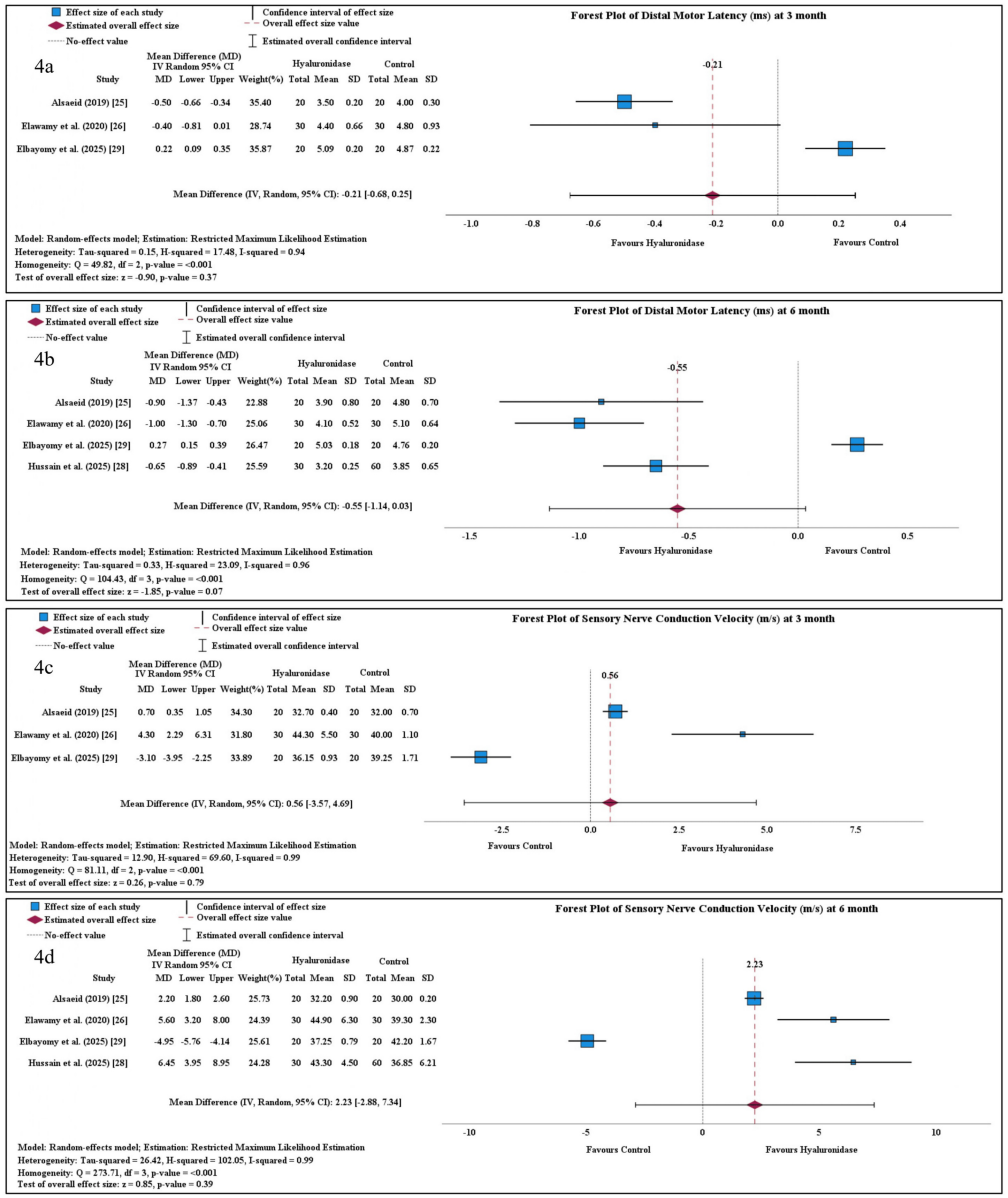
Forest plots comparing hyaluronidase with control interventions for distal motor latency and sensory nerve conduction velocity at three and six months Forest plots comparing hyaluronidase injections with control interventions for electrophysiological outcomes using random-effects meta-analysis. (a) Forest plot for distal motor latency at three months. (b) Forest plot for distal motor latency at six months. (c) Forest plot for sensory nerve conduction velocity at three months. (d) Forest plot for sensory nerve conduction velocity at six months. MD: mean difference; CI: confidence interval; IV: inverse variance; SD: standard deviation

Details of the analysis of DML and SNCV at three and six months are shown in the forest plot in Figure 4.

VAS and CSA of the Median Nerve

The VAS was reported by two RCTs at both three- and six-month follow-ups evaluating the effect of hyaluronidase injection compared with control interventions. At three months, the pooled estimate favored hyaluronidase, but the effect did not reach statistical significance (MD=-1.28; 95% CI=-2.65 to 0.09; p=0.07) and was accompanied by considerable heterogeneity (I^2^=91%; τ²=0.89). At six months, the pooled analysis demonstrated a statistically significant reduction in VAS favoring hyaluronidase (MD=-1.97; 95% CI=-3.88 to -0.06; p=0.04). Heterogeneity was considerable at both three-month (I^2^=91%; τ²=0.89) and six-month (I^2^=91%; τ²=1.74) follow-ups.

**Figure 5:**
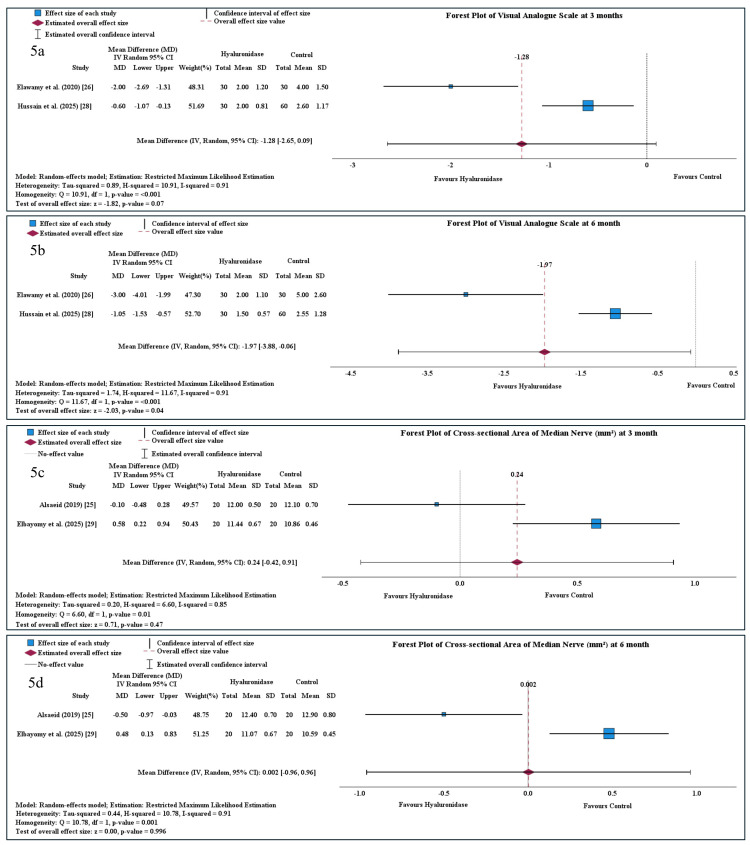
Forest plots comparing hyaluronidase with control interventions for visual analogue scale and median nerve cross-sectional area at three and six months Forest plots comparing hyaluronidase injections with control interventions for pain and ultrasonographic outcomes using random-effects meta-analysis. (a) Forest plot for visual analogue scale scores at three months. (b) Forest plot for visual analogue scale scores at six months. (c) Forest plot for cross-sectional area of the median nerve at three months. (d) Forest plot for cross-sectional area of the median nerve at six months. MD: mean difference; CI: confidence interval; IV: inverse variance; SD: standard deviation

The CSA of the median nerve was reported in two RCTs. The heterogeneity was substantial, and no statistical difference was observed between the hyaluronidase and control groups at both three months (MD=0.24 mm^2^; 95% CI=-0.42 to 0.91; p=0.47) and six months (MD=0.002 mm^2^; 95% CI=-0.96 to 0.96; p=0.996). Details of the pooled analysis of VAS and CSA of the median nerve at three and six months are shown in the forest plot in Figure 5.

Subgroup Analysis: Hyaluronidase vs. Dexamethasone

**Figure 6:**
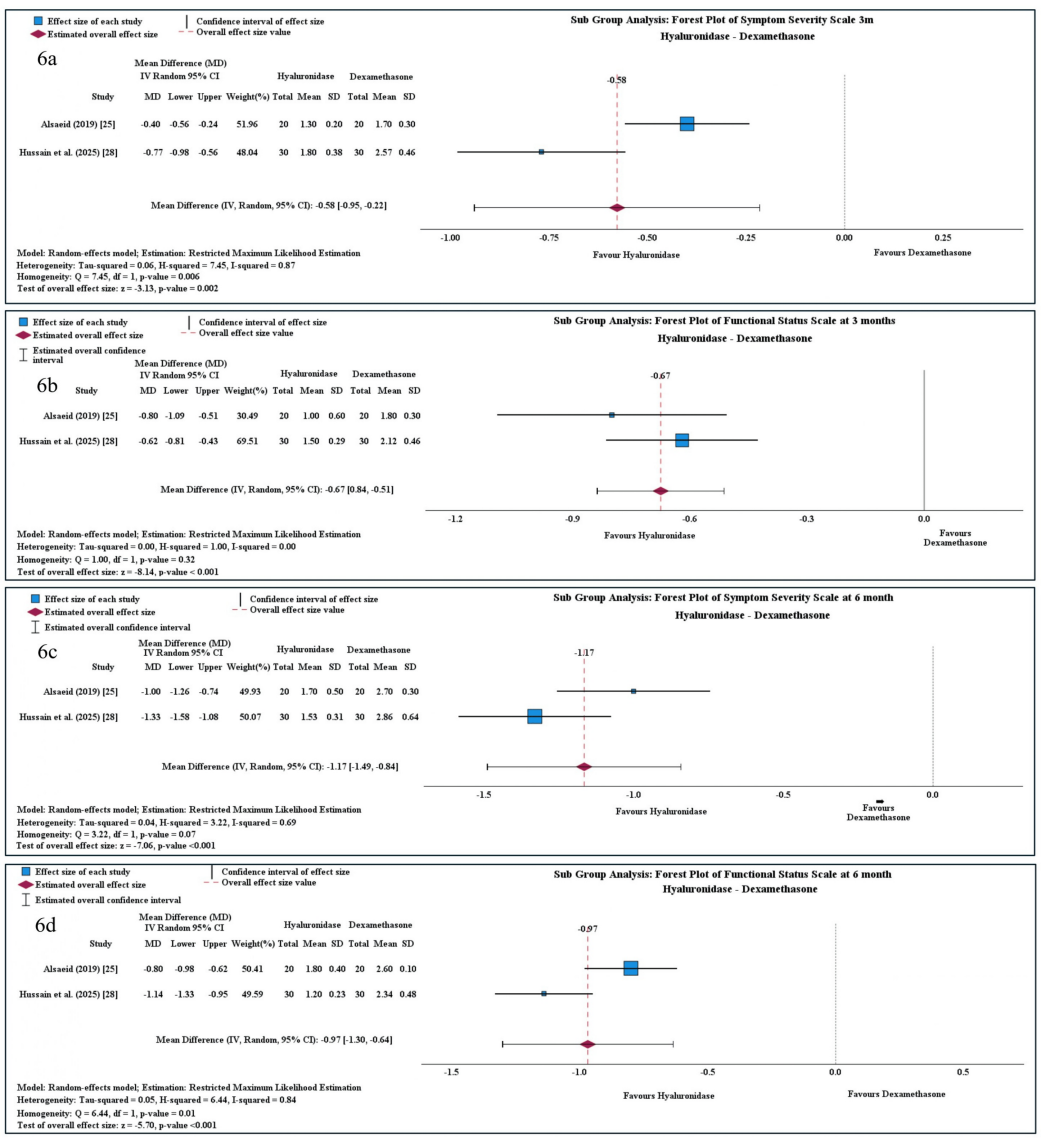
Forest plots of subgroup analysis comparing hyaluronidase with dexamethasone for symptom severity score and functional status scale at three and six months Forest plots of subgroup analysis comparing hyaluronidase injections with dexamethasone for Boston Carpal Tunnel Questionnaire outcomes using random-effects meta-analysis. (a) Forest plot of subgroup analysis for symptom severity score at three months. (b) Forest plot of subgroup analysis for functional status scale at three months. (c) Forest plot of subgroup analysis for symptom severity score at six months. (d) Forest plot of subgroup analysis for functional status scale at six months. MD: mean difference; CI: confidence interval; IV: inverse variance; SD: standard deviation

At three months, the pooled analysis showed a statistically significant reduction in SSS (MD=-0.58; 95% CI=-0.95 to -0.22; p=0.002) in favor of hyaluronidase with substantial heterogeneity (I^2^=87%; τ²=0.06). At three months, FSS showed a significantly favored (MD=-0.67; 95% CI=-0.84 to -0.51; p<0.001) hyaluronidase with no detected heterogeneity (I^2^=0%; τ²=0). At six months, the pooled analysis again favored hyaluronidase in both SSS and FSS but with heterogeneity as shown in Figure 6.

**Figure 7:**
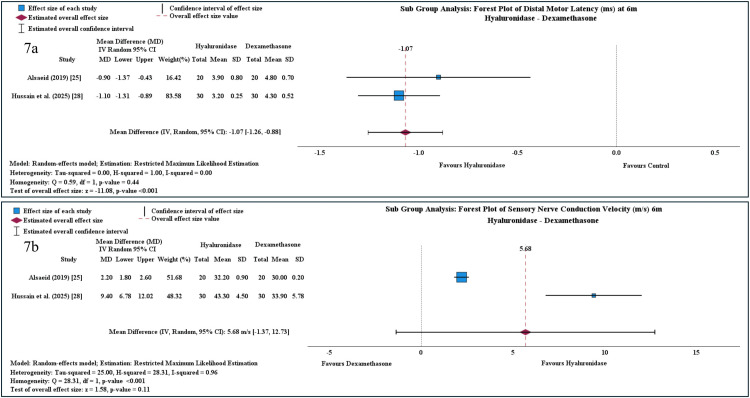
Forest plots of subgroup analysis comparing hyaluronidase with dexamethasone for distal motor latency and sensory nerve conduction velocity at six months Forest plots of subgroup analysis comparing hyaluronidase injections with dexamethasone for electrophysiological outcomes using random-effects meta-analysis. (a) Forest plot of the subgroup analysis for distal motor latency at six months. (b) Forest plot of the subgroup analysis for sensory nerve conduction velocity at six months. MD: mean difference; CI: confidence interval; IV: inverse variance; SD: standard deviation

At six months, the pooled analysis demonstrated significantly greater improvement in DML with hyaluronidase (MD=-1.07; 95% CI=-1.26 to -0.88; p<0.001) and with no heterogeneity (I^2^=0%; τ²=0). For SNCV, there was no statistical difference between hyaluronidase and dexamethasone, as shown in the forest plot in Figure 7.

Subgroup Analysis: Hyaluronidase vs. 5% Dextrose

**Figure 8:**
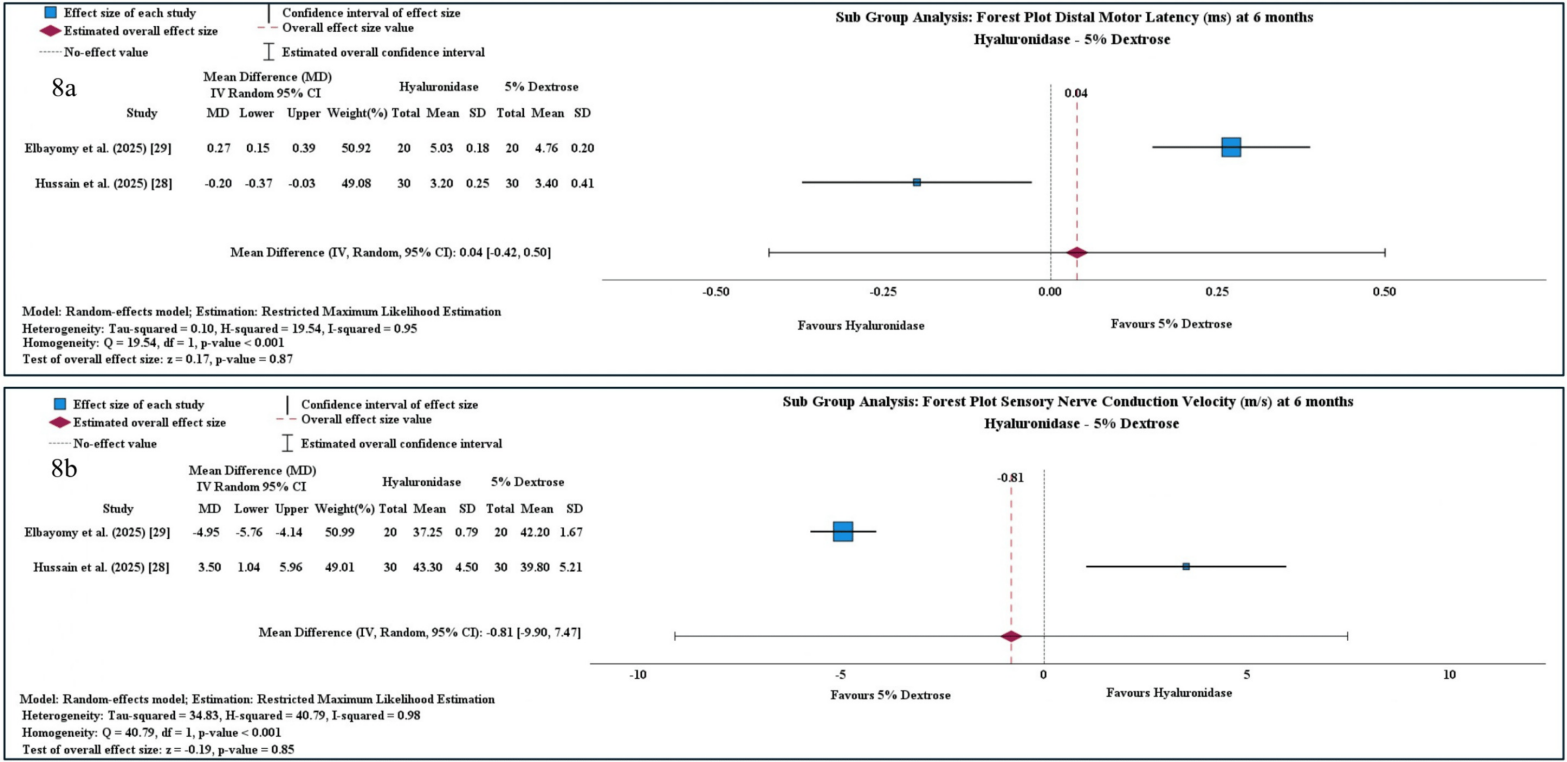
Forest plots of subgroup analysis comparing hyaluronidase with 5% dextrose for distal motor latency and sensory nerve conduction velocity at six months Forest plots of subgroup analysis comparing hyaluronidase injections with 5% dextrose for electrophysiological outcomes using random-effects meta-analysis. (a) Forest plot of the subgroup analysis for distal motor latency at six months. (b) Forest plot of the subgroup analysis for sensory nerve conduction velocity at six months. MD: mean difference; CI: confidence interval; IV: inverse variance; SD: standard deviation

At six months, no statistically significant difference was observed in electrophysiological outcomes. Both analyses showed considerable heterogeneity indicating variability across studies. Details of the analysis are shown in the forest plot in Figure 8.

Sensitivity Analysis

Sensitivity analyses of primary outcomes were performed to evaluate the robustness of pooled estimates by examining the influence of individual RCTs on effect size and heterogeneity. A leave-one-out approach was used for primary outcomes at both three-month and six-month follow-ups.

Sensitivity analysis of FSS at both three and six months demonstrated non-significant pooled effects with considerable heterogeneity. However, exclusion of Elbayomy et al.'s study [29] resulted in statistically significant improvement favoring hyaluronidase at three months (MD=-0.56; 95% CI=-0.99 to -0.13; p=0.01) (I^2^=84%; τ²=0.08) and six months (MD=-0.74; 95% CI=-0.89 to -0.59; p<0.001) (I^2^=10%; τ²=0). Inclusion of this study consistently resulted in loss of significance and persistent heterogeneity.

Sensitivity analysis of SSS at both three and six months showed non-significant pooled effects with considerable heterogeneity. However, exclusion of Elbayomy et al.'s study [29] resulted in a statistically significant improvement favoring hyaluronidase with markedly reduced heterogeneity at both three months (MD=-0.41; 95% CI=-0.54 to -0.29; Z=-6.40; p<0.001) (I^2^=0; τ²=0) and six months (MD=-0.87; 95% CI=-1.15 to -0.6; Z=6.28; p<0.001) (I^2^=46%; τ²=0.02). Inclusion of this study consistently produced non-significant pooled results with persistent heterogeneity.

Sensitivity analysis of DML at three months showed no statistically significant difference except when Elbayomy et al.'s study [29] was excluded, which resulted in a statistically significant result in favor of hyaluronidase (MD=-0.49 ms; 95% CI=-0.63 to -0.34; Z=-6.48; p<0.001) with zero heterogeneity (I^2^=0%; τ²=0). At six months, exclusion of Elbayomy et al.'s study [29] resulted in a statistically significant effect favoring hyaluronidase (MD=-0.83; 95% CI=-1.07 to -0.58; Z=-6.59; p<0.001) (I^2^=44%; τ²=0.02). In the sensitivity analysis of SNCV, exclusion of individual studies resulted in substantial shifts in the direction of effect, some favoring hyaluronidase and some favoring control at both follow-ups.

Certainty of Evidence

The certainty of evidence was assessed using the GRADE framework for four critical outcomes at six months. All four outcomes, that is, SNCV (MD=2.23 m/s; 95% CI=-2.88 to 7.34; p=0.39), SSS (MD=-0.42; 95% CI=-1.29 to 0.45; p=0.35), DML (MD=-0.55 ms; 95% CI=-1.14 to 0.03; p=0.07), and FSS (MD=-0.3; 95% CI=-1.13 to 0.54; p=0.49), demonstrated consistent directional trends favoring hyaluronidase without achieving statistical significance. The certainty of evidence was rated low for all four outcomes, downgraded by two levels: one for serious inconsistency due to substantial between-study heterogeneity and one for serious imprecision as confidence intervals crossed the null threshold in each case. Our confidence in these effect estimates is therefore limited, and the true effects of hyaluronidase may differ substantially from the current pooled estimates. Details of the certainty of evidence are shown in Table 3.

**Table 3:** GRADE certainty of evidence assessment for primary clinical and electrophysiological outcomes at six months This table summarizes the certainty of evidence for primary outcomes using the GRADE approach. Certainty ratings were based on the assessment of inconsistency, indirectness, and imprecision across the included RCTs. Explanations: High certainty: We are very confident that the true effect lies close to that of the estimate of the effect. Moderate certainty: We are moderately confident in the effect estimate: The true effect is likely to be close to the estimate of the effect, but there is a possibility that it is substantially different. Low certainty: Our confidence in the effect estimate is limited: The true effect may be substantially different from the estimate of the effect. Very low certainty: We have very little confidence in the effect estimate: The true effect is likely to be substantially different from the estimate of effect. GRADE: Grading of Recommendations Assessment, Development and Evaluation; RCT: randomized controlled trial; CI: confidence interval; MD: mean difference; SNCV: sensory nerve conduction velocity; BCTQ: Boston Carpal Tunnel Questionnaire; SSS: symptom severity scale; DML: distal motor latency; FSS: functional status scale ^a^considerable heterogeneity; ^b^wide CI crossing thresholds

Certainty assessment						No. of patients		Effect	Certainty	Importance
Outcome	No. of studies	Design	Inconsistency	Indirectness	Imprecision	Hyaluronidase	Control	Absolute (95% CI)		
SNCV	4	RCT	Serious^a^	Not serious	Serious^b^	100	130	MD 2.23 m/s higher (2.88 lower to 7.34 higher)	⨁⨁◯◯ Low^a,b^	Critical
BCTQ: SSS	3	RCT	Serious^a^	Not serious	Serious^a,b^	70	100	MD 0.42 points lower (1.29 lower to 0.45 higher)	⨁⨁◯◯ Low^a^	Critical
DML	4	RCT	Serious^a^	Not serious	Serious^b^	100	130	MD 0.55 ms lower (1.14 lower to 0.03 higher)	⨁⨁◯◯ Low^a,b^	Critical
BCTQ: FSS	3	RCT	Serious^a^	Not serious	Serious^a,b^	70	100	MD 0.3 points lower (1.13 lower to 0.54 higher)	⨁⨁◯◯ Low^a,b^	Critical

None of the included RCTs reported serious adverse events attributable to hyaluronidase injection. Alsaeid [25] specifically noted the absence of allergic reactions, and Elawamy et al. [26] performed pre-procedural skin allergy testing in all participants as a precautionary measure. However, adverse events were not systematically assessed using standardized reporting tools in any of the included trials. Consequently, conclusions regarding the safety profile of hyaluronidase for CTS remain limited.

Discussion

This systematic review and meta-analysis synthesized evidence from four RCTs evaluating the efficacy of hyaluronidase injection in the management of mild to moderate CTS. The findings of the meta-analysis suggest the potential therapeutic relevance of hyaluronidase for mild to moderate CTS.

In the primary meta-analysis, hyaluronidase did not demonstrate clear or consistent improvements in SSS and FSS at both three and six months. Although the direction of effect mostly favored hyaluronidase, DML and SNCV consistently demonstrated directional improvement in favor of hyaluronidase but without reaching statistical significance in pooled analyses and with considerable heterogeneity. Although both clinical and electrophysiological outcomes generally favored hyaluronidase, the limited number of studies and substantial between-study variability preclude definitive conclusions regarding the relative magnitude of improvement across outcomes.

Subgroup analyses demonstrated that hyaluronidase provided significantly greater improvement in FSS, SSS, and DML as compared to dexamethasone. These findings indicate that hyaluronidase may reflect a mechanistically distinct influence on median nerve physiology, likely mediated through enhanced perineural tissue permeability and the restoration of nerve gliding rather than the short‑term modulation of inflammation alone [40,41]. Sensitivity analysis highlighted the limited robustness of findings. The pooled estimates were influenced only by Elbayomy et al.'s study [29]. Exclusion of this study resulted in more consistent findings favoring hyaluronidase.

Ultrasound guidance is an important component of median nerve injections or hydrodissection. It allows the real-time visualization of structures and enables precise needle placement and controlled spread of injectate while minimizing the risk of injury to nerve and other soft tissues [42]. Many studies have shown that ultrasound-guided injections in CTS have significantly better improvement in function and nerve conduction parameters as compared to injections given by palpatory methods [43,44].

In this systematic review and meta-analysis, ultrasonographic evaluation of median nerve CSA did not demonstrate significant reductions at available follow-ups. Structural changes may lag symptomatic improvement [3]. Pain reduction (VAS) showed trends in favor of hyaluronidase and became statistically significant at six months, suggesting a possible delayed analgesic effect of hyaluronidase. Notably, hyaluronidase demonstrated a statistically significant reduction in pain at six months compared with comparator interventions. The pooled effect size (MD=-1.97) approximates the commonly accepted minimal clinically important difference for chronic pain outcomes, suggesting that the observed benefit may be clinically meaningful [45]. However, this finding was based on only two studies and was accompanied by substantial heterogeneity, warranting cautious interpretation.

Considerable heterogeneity observed across all primary outcomes warrants careful consideration. Several sources of clinical and methodological heterogeneity are identifiable. Most importantly, comparator interventions differed substantially across trials. Dexamethasone, normal saline, and 5% dextrose represent injectants with markedly different mechanisms and established efficacy profiles [16,22-24], and pooling them as a single control group inevitably introduces variability in observed treatment differences. Hyaluronidase doses also varied across the included studies, and a dose-response relationship for hyaluronidase in perineural injection has not been established [25,26,28,29].

Additionally, total injectate volume ranged from 5 mL to 10 mL across trials [25,26,28,29], which is clinically significant given that volume itself independently influences the extent of hydrodissection and perineural separation achieved, regardless of the pharmacological agent administered, a phenomenon demonstrated by Evers et al., who showed that saline hydrodissection alone reduces gliding resistance of the median nerve within the carpal tunnel [41]. Elbayomy et al.'s study [29] was identified as the principal driver of observed heterogeneity across all outcomes, and its exclusion yielded markedly reduced heterogeneity and statistically significant pooled effects, suggesting that heterogeneity in comparator efficacy rather than true variability in hyaluronidase effect is the predominant source of inconsistency in this review.

The most common side effect of hyaluronidase reported in the literature is an allergic reaction, which appears more frequently with higher doses [46]. In the included trials, precautionary measures were undertaken, including pre-procedural skin allergy testing or post-injection monitoring. However, adverse events were not consistently evaluated using standardized reporting tools across all included studies [25,26,28,29]. Therefore, while hyaluronidase appears to have a favorable safety profile based on the available data, the evidence is limited and should be interpreted with caution.

In terms of comparative safety, corticosteroid injection, despite its widespread use in CTS, carries well-recognized risks including tendon atrophy, subcutaneous fat atrophy, and systemic glycemic effects, which are particularly problematic in patients with diabetes mellitus [5,16]. In this context, hyaluronidase demonstrated statistically significant improvement over dexamethasone in both patient-reported and electrophysiological outcomes in subgroup analyses, and improvement in DML at six months was achieved with no observed heterogeneity, further strengthening the reliability of this finding. Although hyaluronidase avoids potential corticosteroid-related adverse effects and demonstrated favorable efficacy in several comparisons, the limited number of studies, low certainty of evidence, and inconsistent adverse event reporting preclude definitive conclusions regarding comparative benefit-risk profiles.

In terms of clinical implications, hyaluronidase appears to be a potentially promising therapeutic option for mild to moderate CTS, particularly for patients who require sustained symptom relief or have contraindications to corticosteroid injections. However, given the variability in electrophysiological outcomes and the overall low certainty of evidence, it should currently be considered a viable adjunct or alternative rather than a replacement for established treatments, pending results of larger and more rigorously designed trials.

The overall methodological quality of the included trials was acceptable with no study classified as high risk. The certainty of evidence was rated as low for key outcomes. This was primarily due to substantial inconsistency across studies and the imprecision of effect estimates, with confidence intervals crossing the line of no effect. Consequently, the true effect of hyaluronidase may differ substantially from the current pooled estimates, and further high-quality research is likely to have an important impact on these findings.

This review has several strengths, including the inclusion of only RCTs, the use of standardized outcome measures, and the application of subgroup and sensitivity analyses to assess the robustness of findings.

Limitations

Despite potentially promising findings, limitations exist. The total number of studies is only four, the total sample size across studies remains modest, and follow-up periods did not exceed six months, leaving long-term durability uncertain. Most analyses had considerable heterogeneity. This heterogeneity may be due to variability in comparators, volume, and dose of injectate reflecting the diversity across the included trials.

## Conclusions

Current evidence from four RCTs does not demonstrate consistent statistically significant superiority of hyaluronidase-based injections over comparator interventions for clinical or electrophysiological outcomes in mild to moderate CTS. Although pooled estimates generally favored hyaluronidase, substantial heterogeneity and the small evidence base limit confidence in these findings. Subgroup analyses suggested potential benefits over dexamethasone-based hydrodissection; however, these results should be interpreted cautiously. No major safety concerns were identified, but safety data were limited and inconsistently reported. Given the overall low certainty of evidence and the presence of some risk of bias concerns among the included studies, further adequately powered trials with standardized protocols, uniform hyaluronidase dosing, detailed adverse reaction reporting, and longer follow-up are required to clarify the effectiveness and safety of hyaluronidase-assisted hydrodissection.

## Appendices

Electronic search strategy

PubMed: All fields (((carpal tunnel syndrome) OR (CTS)) OR (Median Nerve Entrapment) AND (1000/1/1:2025/7/31[pdat])) AND ((Hyalase) OR (Hyaluronidase) AND (1000/1/1:2025/7/31[pdat])) 

Scopus: All fields ("Carpal Tunnel Syndrome" OR CTS OR "Median Nerve Entrapment") AND ("Hyaluronidase" OR "Hyalase")

Cochrane Central Register of Controlled Trials: (("carpal tunnel syndrome"):ti,ab,kw OR (CTS):ti,ab,kw OR (Median Nerve Entrapment):ti,ab,kw) AND (("hyaluronidase"):ti,ab,kw OR (Hyalase):ti,ab,kw)
